# Integrated Human-Animal Disease Surveillance

**DOI:** 10.3201/eid1109.050180

**Published:** 2005-09

**Authors:** Whitney A. Mauer, John B. Kaneene

**Affiliations:** * Michigan State University, East Lansing, Michigan, USA

**Keywords:** needs assessment, veterinarian, zoonoses, infectious diseases, emerging, disease notification, computer systems, surveillance, letter

**To the Editor:** Early identification of zoonotic disease occurrence through simultaneous monitoring of human and animal disease surveillance systems is critical to protect health in both populations. We assessed the surveillance and reporting needs of a small but diverse group of Michigan veterinarians by examining their perspective of the current animal disease reporting system, the system enhancements desired, and their computer and Internet accessibility.

Developing systems that link human and animal disease reporting systems can help identify and facilitate a response to known and emerging zoonotic diseases. A system that allows simultaneous electronic capture and assessment of human and animal disease reports is being implemented in Michigan. The system will be based on the Michigan Disease Surveillance System platform, a Web-based human disease reporting system implemented by the Michigan Department of Community Health (*1**,**2*).

To ensure an integrated system that meets both human and veterinary public health needs, we developed a questionnaire for veterinarians to collect information on the current animal disease reporting system, system enhancements that are desired, and computer and Internet access capabilities (University Human Research Committee approved). In July 2003, a total of 112 questionnaires was sent to a convenience sample of Michigan veterinarians who represent a variety of practice types and sizes, participate in organized veterinary medicine and academia, and would be motivated to participate in system development. Of the 112 questionnaires, 84 (75%) were completed. Of 79 practices represented, 19 (24%) treated companion animals, 15 (19%) treated equids, 4 (5%) treated food animals (dairy, beef, small ruminant, poultry, or swine), 32 (41%) treated a variety of animals (no patient type >75%), 4 (5%) treated zoo animals, and 5 (6%) veterinarians were not in clinical practice.

Of 81 respondents, 75 (93%) indicated that they were aware of the Michigan reportable animal disease list. Yet, 37 (47%) of 79 respondents have reported no cases and 32 (41%) of 79 have reported 1 to 5 cases annually. Furthermore, only 19 (25%) of 76 respondents reported that their clients might have confidentiality issues that could impede disease reporting. Therefore, it will be important to continue educating veterinarians to ensure that suspected disease cases are reported as well as confirmed cases, which may have been reported by the laboratory also.

Positive feedback was received regarding the current animal disease reporting system. Respondents evaluated the availability of a published list of reportable diseases (68 [89%] of 76) and the availability and quality of laboratory testing and disease confirmation (57 [75%] of 76) as Strongly Like/Like (Scale = Strongly Dislike, Dislike, No Opinion, Like, Strongly Like). However, case submission feedback may need to be assessed as only 34 (45%) of 76 respondents evaluated this service as Strongly Like/Like and 13 (17%) of 76 evaluated it as Strongly Dislike/Dislike.

Of 73 respondents, 68 (93%) reported that routinely published animal disease surveillance data would be beneficial for themselves or their clients. Of 55 respondents, 24 (44%) indicated that these data would be used to increase disease awareness and 23 (42%) indicated the data would be used for risk assessment and prevention planning. Outbreak alert information, easy access to regulatory requirements, and animal disease information were also desired.

Virtually all practices had a computer (79 [99%] of 80), Internet access (70 [88%] of 80), and email (53 [75%] of 71). Most (68 [91%] of 75) were comfortable using a Web-based application to submit case reports. However, because Internet access and email may not be universal, multiple modes of communication must be utilized. Furthermore, of 81 respondents, 29 (36%) indicated lack of time, and 24 (30%) indicated lack of staff as a barrier to online reporting. Therefore, the reporting system should be efficient.

Rabies, bovine tuberculosis, and West Nile virus, all zoonotic diseases, were listed by >50% of the 79 respondents as 1 of the 6 diseases they felt were most important in Michigan. Simultaneous tracking of human and animal diseases was considered useful by 32 (84%) of 38 respondents because animals are sentinels of human (zoonotic) disease and by 7 (18%) respondents because of the threat of agroterrorism. In general, respondents are aware of the importance of animal disease reporting to public health.

In Michigan, the human and animal disease reporting systems are similarly structured, although there is no local level animal health agency (Figure). These similarities can provide the basis for a system that is functionally appropriate to track diseases in humans and multiple animal species and meet multiple agency surveillance objectives.

**Figure:**
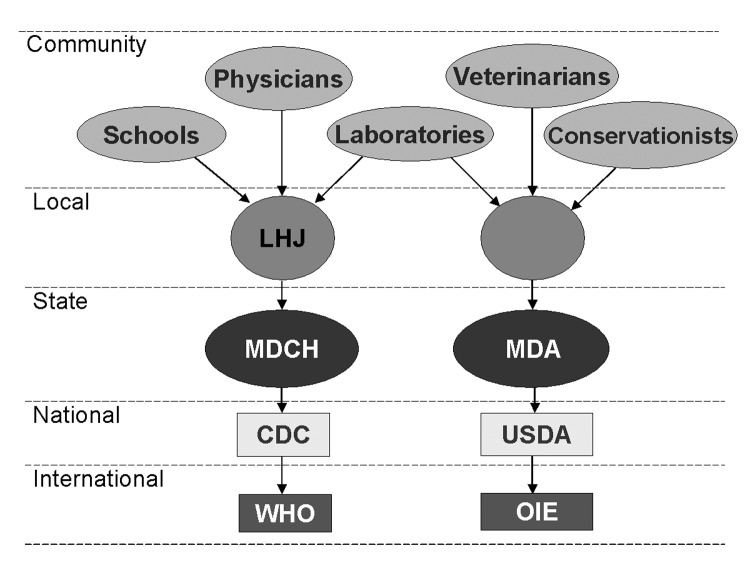
Comparison of Michigan human and animal disease reporting system structures. LHJ, Local Health Jurisdiction; MDCH, Michigan Department of Community Health; CDC, Centers for Disease Control and Prevention; WHO, World Health Organization; MDA, Michigan Department of Agriculture, Animal Plant Health Inspection Service, Veterinary Services; USDA, US Department of Agriculture; OIE, Office International Epizooties.

Overall, this group of Michigan veterinarians considers developing a Web-based disease reporting system as useful as long as the following issues are addressed: 1) quality case report feedback; 2) access to correct and coordinated human and animal disease information; and 3) computer system reliability and efficiency. Based on the results of this study, the second phase of this project, construction of the animal disease surveillance portion of Michigan's reporting system, will be implemented with continued input from local, state, and federal stakeholders.
